# Lung aeration estimated by chest electrical impedance tomography and lung ultrasound during extubation

**DOI:** 10.1186/s13613-023-01180-3

**Published:** 2023-09-26

**Authors:** Vincent Joussellin, Vincent Bonny, Savino Spadaro, Sébastien Clerc, Mélodie Parfait, Martina Ferioli, Antonin Sieye, Yorschua Jalil, Vincent Janiak, Andrea Pinna, Martin Dres

**Affiliations:** 1 Sorbonne Université, INSERM, UMRS1158 Neurophysiologie Respiratoire Expérimentale et Clinique, Paris, France; 2 Hôpital Pitié-Salpêtrière, Service de Médecine Intensive, Réanimation (Département “R3S”), AP-HP, Sorbonne Université, 47‑83 boulevard de l’Hôpital, 75013 Paris, France; 3 Department of Translational Medicine, Intensive Care Unit, University of Ferrara, Sant’Anna Hospital, Ferrara, Italy; 4 Sorbonne Université, CNRS, LIP6, 75005 Paris, France; 5 Bioserenity, 20 Rue Berbier-Du-Metz, 75013 Paris, France; 6 Respiratory and Critical Care Unit, IRCCS Azienda Ospedaliero-Universitaria di Bologna, Bologna, Italy; 7 Department of Clinical, Integrated and Experimental Medicine (DIMES), Alma Mater Studiorum University of Bologna, Bologna, Italy; 8 Departamento de Medicina Intensiva, Facultad de Medicina, Pontificia Universidad Católica de Chile, Santiago, Chile; 9 Departamento de Ciencias de la Salud, Carrera de Kinesiología, Facultad de Medicina, Pontificia Universidad Católica de Chile, Santiago, Chile

**Keywords:** Electrical impedance tomography, Lung ultrasound, Mechanical ventilation, Extubation failure, Weaning

## Abstract

**Background:**

This study hypothesized that patients with extubation failure exhibit a loss of lung aeration and heterogeneity in air distribution, which could be monitored by chest EIT and lung ultrasound. Patients at risk of extubation failure were included after a successful spontaneous breathing trial. Lung ultrasound [with calculation of lung ultrasound score (LUS)] and chest EIT [with calculation of the global inhomogeneity index, frontback center of ventilation (CoV), regional ventilation delay (RVD) and surface available for ventilation] were performed before extubation during pressure support ventilation (H0) and two hours after extubation during spontaneous breathing (H2). EIT was then repeated 6 h (H6) after extubation. EIT derived indices and LUS were compared between patients successfully extubated and patients with extubation failure.

**Results:**

40 patients were included, of whom 12 (30%) failed extubation. Before extubation, when compared with patients with successful extubation, patients who failed extubation had a higher LUS (19 vs 10, *p* = 0.003) and a smaller surface available for ventilation (352 vs 406 pixels, *p* = 0.042). After extubation, GI index and LUS were higher in the extubation failure group, whereas the surface available for ventilation was lower. The RVD and the CoV were not different between groups.

**Conclusion:**

Before extubation, a loss of lung aeration was observed in patients who developed extubation failure afterwards. After extubation, this loss of lung aeration persisted and was associated with regional lung ventilation heterogeneity.

*Trial registration* Clinical trials, NCT04180410, Registered 27 November 2019—prospectively registered, https://clinicaltrials.gov/ct2/show/NCT04180410.

**Supplementary Information:**

The online version contains supplementary material available at 10.1186/s13613-023-01180-3.

## Background

The most severe forms of acute respiratory failure (ARF) require invasive mechanical ventilation to ensure the viability of gas exchange. Several cohort studies have pointed out the negative impact of prolonged mechanical ventilation on survival and disability [1, 2]. It is therefore needed to liberate the patients from the ventilator as soon as they are ready for. However, untimely separation from the ventilator increases the risk of extubation failure which occurs in 10–25% of the patients [3, 4] and is associated with significant morbidity and mortality [5]. Delay between extubation and reintubation is associated with mortality [6]. Accordingly, close monitoring of patients at risk of extubation failure may be useful to decide when to implement noninvasive respiratory supports [6]. Nevertheless, early detection of respiratory deterioration is not straightforward after extubation since the ventilator signals are no more available. A continuous and noninvasive monitoring of the respiratory function in spontaneously breathing patients would be of great value. The interest of lung ultrasound has been demonstrated to document loss of lung aeration during the spontaneous breathing trial (SBT) in patients who subsequently had failed extubation [7]. When compared with lung ultrasound, electrical impedance tomography (EIT) is a continuous, noninvasive, nonoperator-dependent imaging technique of regional lung ventilation [8]. Hence, assessing the regional lung ventilation with EIT could provide a continuous and noninvasive measurement of lung derecruitment [9] and indicate early warning signals in patients who could require initiation of preventive strategies. The present study hypothesized that loss of lung aeration and air distribution heterogeneity could be monitored by chest EIT and lung ultrasound in patients with extubation failure. Therefore, the objectives of the present study were to describe the regional lung ventilation with chest EIT and lung ultrasound of patients before and after extubation, and to compare them according to extubation failure or success.

## Methods

We conducted a prospective observational study in a medical intensive care unit of Pitié-Salpetrière Hospital (Assistance Publique—Hôpitaux de Paris) from February 2020 to May 2022. This study complies with the Strengthening the Reporting of Observational Studies in Epidemiology (STROBE) Statement. The study was approved by the Comité de Protection des Personnes du Nord Ouest I (N° ID-RCB: 2019-A02986-51) and has been performed in accordance with the ethical standards laid down of the 2008 Declaration of Helsinki. Written and oral information about the study was given to patients or their families prior to enrolment. Informed consent was obtained from the patients or their relatives. The study was publicly registered on ClinicalTrial.gov (NCT 04180410) prior inclusion of the first patient. The sponsor of the study was Bioserenity SA who had no role in the design of the study.

### Study population

We included patients older than 18 years, who were mechanically ventilated for at least 48 h through an orotracheal tube. They had to present at least one risk factor of extubation failure: age > 65 years old, chronic heart disease, chronic pulmonary diseases. Chronic heart disease included chronic cardiac diseases with left ventricular dysfunction (defined by left ventricular ejection fraction ≤ 45%, whatever the cause), history of cardiogenic pulmonary edema, documented ischemic heart disease, or permanent atrial fibrillation. Chronic pulmonary diseases included chronic obstructive pulmonary disease, obesity-hypoventilation syndrome, or restrictive pulmonary disease. In addition, patients had to succeed to a spontaneous breathing trial (see below). Noninclusion criteria were pregnancy, contra indications to EIT (chest tube, cardiac pacemaker or implanted defibrillator, cervical implants) and the use of extracorporal assistance (ECMO).

### Weaning protocol

According to the institutional weaning protocol, before starting a spontaneous breathing trial (SBT), patients had to meet predefined readiness-to-wean criteria on daily screening [10]. The SBT was conducted without any kind of ventilatory support (pressure support and PEEP set to 0 cmH_2_O), for at least 30 min, while FiO_2_ remained unchanged. SBT failure was defined by one of the following criteria: respiratory rate ≥ 35/min or increase ≥ 50%, SpO_2_ ≤ 90% or PaO_2_ ≤ 50 mmHg (with FiO_2_ ≥ 50%), heart rate ≥ 140 bpm, de novo supraventricular or ventricular arrhythmia, systolic arterial pressure > 180 or < 90 mmHg, alteration of consciousness, diaphoresis, or any signs of respiratory distress. In case of SBT success, patients were considered ready to be extubated by the physician in charge and were approached for inclusion by the investigators. The preventive use of high-flow nasal oxygen (HFNO) and/or noninvasive ventilation (NIV) after extubation was based on the presence of predefined risk factors of extubation failure [11, 12], it was not intended to be used to treat patients presenting with postextubation respiratory distress. Introduction and duration of HFNO and/or NIV sessions were let at the discretion of the clinicians.

### Data collection and study protocol

Upon inclusion, demographic data were prospectively collected: age, gender, comorbidities, date of intensive care unit admission, date of intubation, main reason for intubation, ventilator settings, weight and height upon admission and fluid balance over the last 24 h of the inclusion. Before extubation, arterial blood gases, lactate, plasma protein concentration, hemoglobin were sampled. In addition, the following clinical variables were prospectively collected before and after extubation: systolic and diastolic arterial pressure, heart rate, respiratory rate, SpO_2_, HFNO or NIV settings if currently used. At each study’s visit [i.e., before extubation (H0), two hours after extubation (H2) and 6 h after extubation (H6)], clinical and laboratory variables were collected and two 5-min EIT measurements were recorded. Lung ultrasound (to evaluate lung aeration) and echocardiography (to assess left ventricular function and cardiac filling pressures) were performed at H0 and H2 (not at H6). Cardiac function was evaluated before extubation (H0) and 2 h (H2) after extubation to collect the following variables: left ventricular ejection fraction (visual estimation), early (E) and late (A) diastolic wave velocities at the mitral valve, tissue Doppler early (e’) wave velocity at the lateral mitral valve annulus, cardiac output as estimated by the stroke volume measured using the Doppler method applied at the left ventricular outflow tract.

### EIT measurements

After enrolment, patients were placed in a semi-recumbent position. A silicon 16-electrode EIT belt of proper size was placed around the patient’s chest between the 4th and 6th intercostal spaces and connected to the EIT device (PulmoVista 500; Draeger Medical GmbH, Lübeck, Germany). Before extubation (H0), EIT was connected to a ventilator (Infinity V500; Drager Medical GmbH, Lübeck, Germany) through a RS232 interface to calibrate impedance variation with volume. EIT recordings were sampled at 30 Hz, downloaded as a file, and analyzed offline on a personal computer using a dedicated software (EITdiag and EITanalysis, Draeger Medical GmbH, Lübeck, Germany). The five more representative minutes over the 10 min recording were analyzed to eliminate artefacts related to patient’s movements or cough. From the EIT recordings, we calculated EIT derived indices:

- The inhomogeneity index [13] (GI) quantified the homogeneity of ventilation distribution. The higher the value, the more heterogeneous the ventilation, with coexistence of regions with low and high impedance variations. It is calculated as follows:
  $${\text{GI}} = \frac{{\sum {_{{{\text{pixel}}{\kern 1pt} {\text{lung}}}} \left[ {\Delta Z_{{{\text{pixel}}}} - {\text{Median}}\left( {\Delta Z_{{{\text{lung}}}} } \right)} \right]} }}{{\sum {_{{{\text{pixel}}{\kern 1pt} {\text{lung}}}} \Delta Z_{{{\text{pixel}}}} } }}$$
  where ${\Delta Z}_{\mathrm{pixel}}$ is the difference between inspiratory and expiratory impedance of a given pixel and ${\Delta Z}_{\mathrm{lung}}$ the difference between inspiratory and expiratory impedance of the whole lung.
- The center of ventilation [14] (CoV) reflects the air distribution. It is an average of the two points that devise each lung in two equal parts of impedance variation in an anterior–posterior axis. Thus, a value of 50% corresponds to a perfectly balanced ventilation. If the posterior part of the lung is poorly ventilated (due to atelectasis for example), the center of ventilation moves anteriorly: the value will be higher than 50%.
- The regional ventilation delay [15] (RVD) is expressed as a percentage of the whole lung. It corresponds to the sum of local pixel in a significant delay during inspiration as compared to the whole lung. Only pixels with an impedance change > 15% of the maximum impedance change are included. A high value suggests an alveolar derecruitment.
- The surface available for ventilation is a novel index provided by the software (EITdiag and EITanalysis, Draeger Medical GmbH, Lübeck, Germany). It represents the number of pixels in which the local impedance change is greater than a predetermined percentage of the maximum of the local impedance change, calculating the ventilated surface area. The higher the value, the greater the surface of ventilated area.

### Lung aeration

After EIT measurements, lung ultrasound (Philips Sparq) was performed by a trained investigator (VJ) before extubation (H0) and 2 h (H2) after extubation. A 2–4 MHz convex probe was used to scan the whole lung of both sides. The number of B-lines was counted on a rib short-axis scan between two ribs at each intercostal space of the upper and lower parts of the anterior, lateral, and posterior regions of the left and right chest wall (total of 12 areas). For a given region of interest, points were allocated according to the worst ultrasound pattern observed [7]: presence of lung sliding with A lines or fewer than two isolated B lines (normal pattern, score 0); multiple, well-defined B line (moderate loss of lung aeration, score 1); multiple coalescent B lines (severe loss of lung aeration, score 2); lung consolidation (score 3). The lung ultrasound score (LUS) is calculated between 0 (normally aerated lung) and 36 (totally consolidated lung) [7].

### Study objectives and endpoints

Initially, our goal was to perform EIT and lung ultrasound until 48 h after extubation. However, due to attrition rate in the failure group (see Additional file 1), it was deemed not possible to perform statistical analysis after H6. Accordingly, the primary objective was redefined to compare regional lung ventilation before extubation and at H2 and H6 after extubation, between patients with an extubation success and patients with an extubation failure. Extubation failure was defined either as death or reintubation within 48 h after extubation or as the reinstitution of any mechanical ventilation after extubation, either invasive or noninvasive, with a curative indication [16]. Indication for reinstitution of mechanical ventilation support was postextubation acute respiratory failure, defined by the presence of one or more of the following criteria (if persistent over 5 min): SpO2 < 90% with an oxygen support ≥ 5 L/min, a respiratory rate ≥ 35/min, a pH < 7.35 with a pCO_2_ > 45 mmHg. The secondary objectives were (1) to describe the time-course evolution of EIT derived indices during the 6 h after extubation, (2) to correlate EIT derived indices with lung ultrasound score.

### Statistical analysis

Owing to the lack of published data at the time of the study design and the exploratory nature of the study, no power analysis was performed. We planned to enroll a convenient sample of 40 patients, to obtain at least 10 patients with extubation failure with an estimated rate of extubation failure of 25%. Continuous data were expressed as median (25th–75th interquartile range). Categorical data were expressed as number (percentage). Comparisons between extubation failure and extubation success were performed with a Wilcoxon rank-sum test, or Weich’s t test in case of normal distribution. The data distribution was assessed by a Shapiro–Wilk test. Categorical data were compared by Fisher’s exact test or Chi-square depending on the sample size. Correlations between EIT derived indices and LUS were analyzed with linear regression and Spearman’s correlation, with all the data from H0 and H2 pooled together. Receiver-operating characteristic (ROC) curves were constructed to evaluate the performance of the LUS and EIT variables to predict extubation failure. Sensitivities, specificities, positive and negative predictive values, and areas under the ROC curves (AUC–ROC) were calculated. The best threshold value for each variable was determined as the value associated with the best Youden index for the prediction of extubation failure. In cases where two thresholds yielded the same Youden index, the threshold with the highest sensitivity was given preference. A two-sided *p* < 0.05 was considered significant. Statistical analyses were performed with R version 4.2.1 (R Development Core Team 2011; R Foundation for Statistical Computing, Vienna, Austria).

## Results

### Study population

**Fig. 1:**
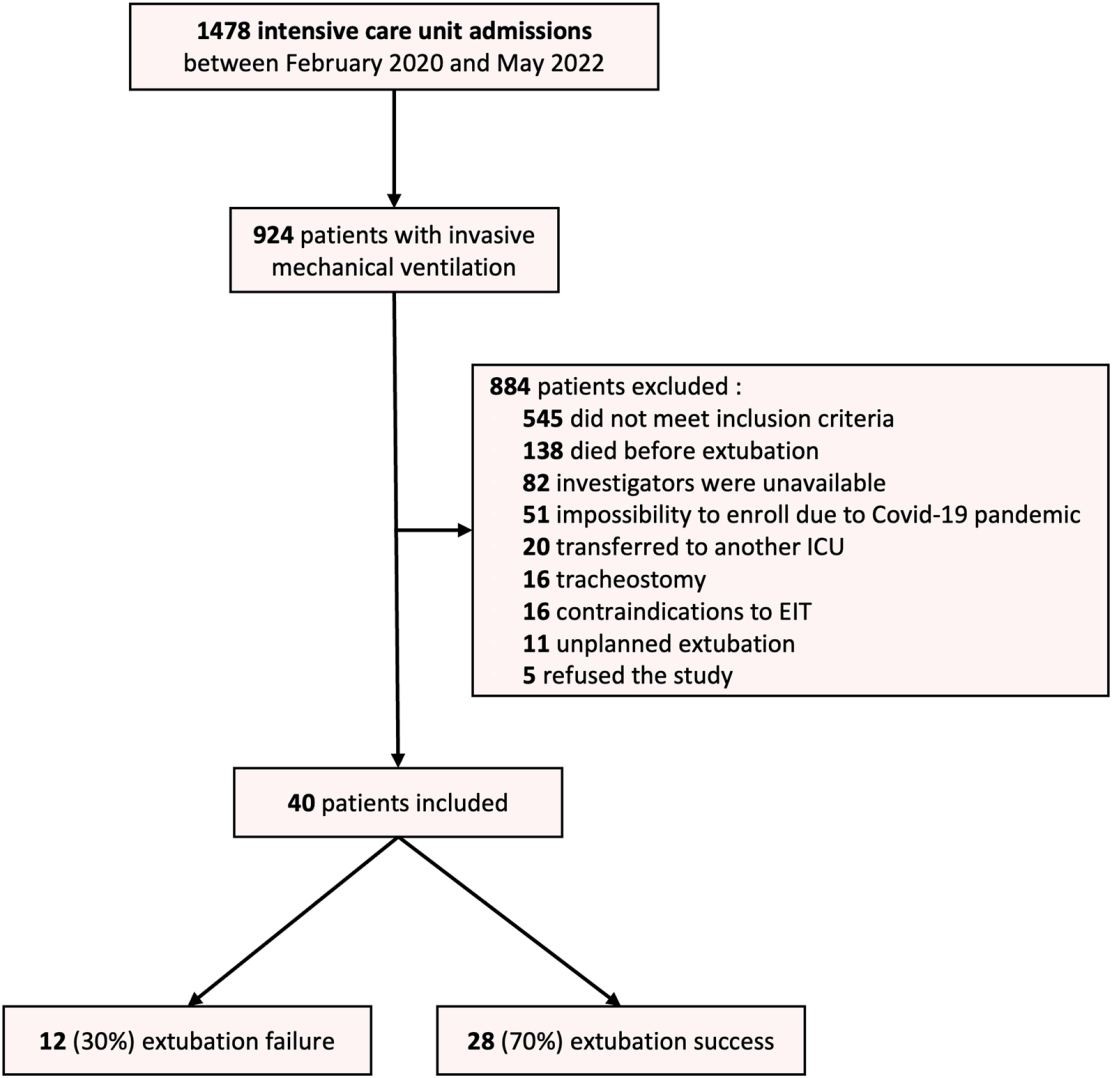
Flowchart of the study

From February 2020 to May 2022, 1478 patients were admitted to ICU, and 924 received mechanical ventilation support. Of these, 884 patients were excluded from the analysis. The causes of noninclusion are shown in the flow chart (Fig. 1). Forty patients were finally included in the study (see Additional file 1).

Table 1 displays the main characteristics of the patients at inclusion. The presence of chronic respiratory disease was the main risk factor for extubation failure. The median duration of mechanical ventilation at the time of extubation was 10 (6–15) days.

**Table 1:** Characteristics of the study population upon inclusion

Characteristics	Overall *N* = 40	Extubation failure *N* = 12	Extubation success *N* = 28	*p*-value
Age, years	59 (48–67)	59 (49–68)	59 (48–65)	0.885
Female sex, *n* (%)	11 (28)	4 (33)	7 (25)	0.877
Body mass index, kg.m^−2^	27 (23–31)	27 (23–30)	27 (23–31)	0.735
24 h fluid balance, L	− 1.1 (− 2.1–0.2)	− 1.0 (− 1.9 to − 0.1)	− 1.3 (− 2.1–0.2)	0.851
SOFA score	8 (4–11)	6 (4–8)	8 (4–12)	0.075
SAPS II	52 (40–66)	43 (36–54)	54 (44–71)	**0.024**
Days of ventilation before extubation	10 (6–15)	10 (7–14)	10 (5–15)	0.871
Extubation failure risk factors, *n* (%)				
Age > 65 years old	12 (30)	4 (33)	8 (29)	1.000
Chronic cardiac disease	17 (42)	4 (33)	13 (46)	0.443
Chronic respiratory disease	24 (60)	5 (42)	19 (68)	0.166
Main reason for intubation, *n* (%)				
Acute respiratory failure	25 (62)	10 (83)	15 (54)	0.154
Cardiac arrest	6 (15)	0 (0)	6 (21)	0.153
Coma	6 (15)	1 (8)	5 (18)	0.648
Shock	2 (5)	1 (8)	1 (4)	0.515
Other	1 (2)	0 (0)	1 (4)	> 0.999
Ventilator settings				
Vt PBW, ml.kg^−1^	7.2 (6.0–8.5)	6.9 (5.6–7.5)	7.5 (6.1–8.8)	0.220
Pressure support, cmH_2_O	10 (8–12)	10 (8–12)	10 (8–12)	0.773
PEEP, cmH_2_O	5 (5–6)	5 (5–6)	6 (5–6)	0.275

*SAPS II* simplified acute physiology score, *SOFA* sepsis related organ failure assessment, *Vt* tidal volume, *PEEP* positive end expiratory pressure

### Weaning outcome

After extubation, prophylactic NIV was used in 23 patients (58%) and high flow nasal oxygen in 8 patients (20%). Extubation failure occurred in 12 (30%) patients within the 48 h after extubation. Among the 12 patients classified as failed extubation, 10 (25%) needed to be re-intubated and two (5%) experienced acute respiratory failure needing curative form of noninvasive respiratory support without being re-intubated. Four (10%) patients finally died in ICU. The median duration between extubation and occurrence of extubation failure was 10 (1–24) hours. The main cause of extubation failure was the presence of ineffective cough (*n* = 6, 50%).

### Respiratory and cardiac function before extubation

Clinical and biological markers, lung ultrasound, echocardiography data, and EIT derived indices upon inclusion are presented in Table 2. By contrast to patients who succeed extubation, those who failed had a lower PaO_2_/FiO_2_ ratio and a higher respiratory rate. The LUS was higher (19 vs 10, *p* = 0.003). The GI index, the CoV and the RVD were not different between groups, while the surface available for ventilation was smaller in patients who developed extubation failure afterwards (352 vs 406 pixels, *p* = 0.042).

**Table 2:** Clinical and biological variables, cardiac function, lung aeration, and EIT derived indices before extubation, according to extubation outcome

Characteristics	Overall *N* = 40	Extubation failure *N* = 12	Extubation success *N* = 28	*p*-value
Clinical variables				
Systolic arterial blood pressure, mmHg	130 (125–140)	130 (126–138)	128 (121–140)	0.627
Heart rate, min^−1^	95 (80–116)	99 (83–120)	94 (79–105)	0.481
Respiratory rate, min^−1^	23 (20–28)	28 (25–30)	22 (18–26)	**0.006**
IC-RDOS	2.37 (2.22–2.83)	2.48 (2.27–2.88)	2.35 (2.20–2.70)	0.570
Laboratory variables				
pH	7.45 (7.43–7.49)	7.48 (7.45–7.50)	7.44 (7.42–7.48)	0.074
PaO_2_/FiO_2_ ratio, mmHg	257 (203–314)	198 (164–214)	290 (245–329)	**< 0.001**
PaCO_2_, mmHg	41 (38–46)	39 (36–41)	42 (39–46)	0.077
Hematocrit, %	27.8 (24.6–31.0)	30.5 (24.7–31.2)	27.6 (24.6–30.2)	0.570
Protein, g.L^−1^	62 (59–68)	64 (61–68)	62 (58–68)	0.665
Cardiac function				
Left ventricle ejection fraction, %	50 (42–51)	50 (48–58)	45 (40–50)	0.106
E/A ratio	1.1 (0.9–1.3)	1.2 (0.8–1.5)	1.1 (0.9–1.2)	0.792
E/E’ ratio	7.5 (6.3–9.9)	8.6 (5.9–10.9)	7.5 (6.6–9.9)	0.973
Cardiac output, L.min^−1^	7.2 (5.9–8.5)	8.2 (6.7–8.9)	7.1 (5.7–8.4)	0.182
Lung aeration				
Lung ultrasound score	12 (8–18)	19 (14–22)	10 (6–15)	**0.003**
Posterior lung ultrasound score	6 (3–8)	8 (7–10)	5 (2–7)	** < 0.001**
Electrical impedance tomography				
Surface, nb of pixels	394 (352–443)	352 (324–399)	406 (382–451)	**0.042**
Global inhomogeneity index	0.38 (0.36–0.40)	0.38 (0.37–0.40)	0.37 (0.36–0.40)	0.718
Center of ventilation (front-back), %	50 (46–52)	50 (47–52)	50 (46–52)	0.766
Regional ventilation delay_,_ %	8.0 (6.6–10.0)	9.1 (7.6–9.7)	7.7 (5.7–10.5)	0.167

IC-RDOS: Intensive care-respiratory distress observation scale; E/A: early (E) over late (A) diastolic wave velocity ratio; E/e’: E wave over tissue Doppler early (e’) wave velocities at the lateral mitral valve annulus

### Time-course evolution of lung ultrasound and EIT derived indices after extubation

**Fig. 2:**
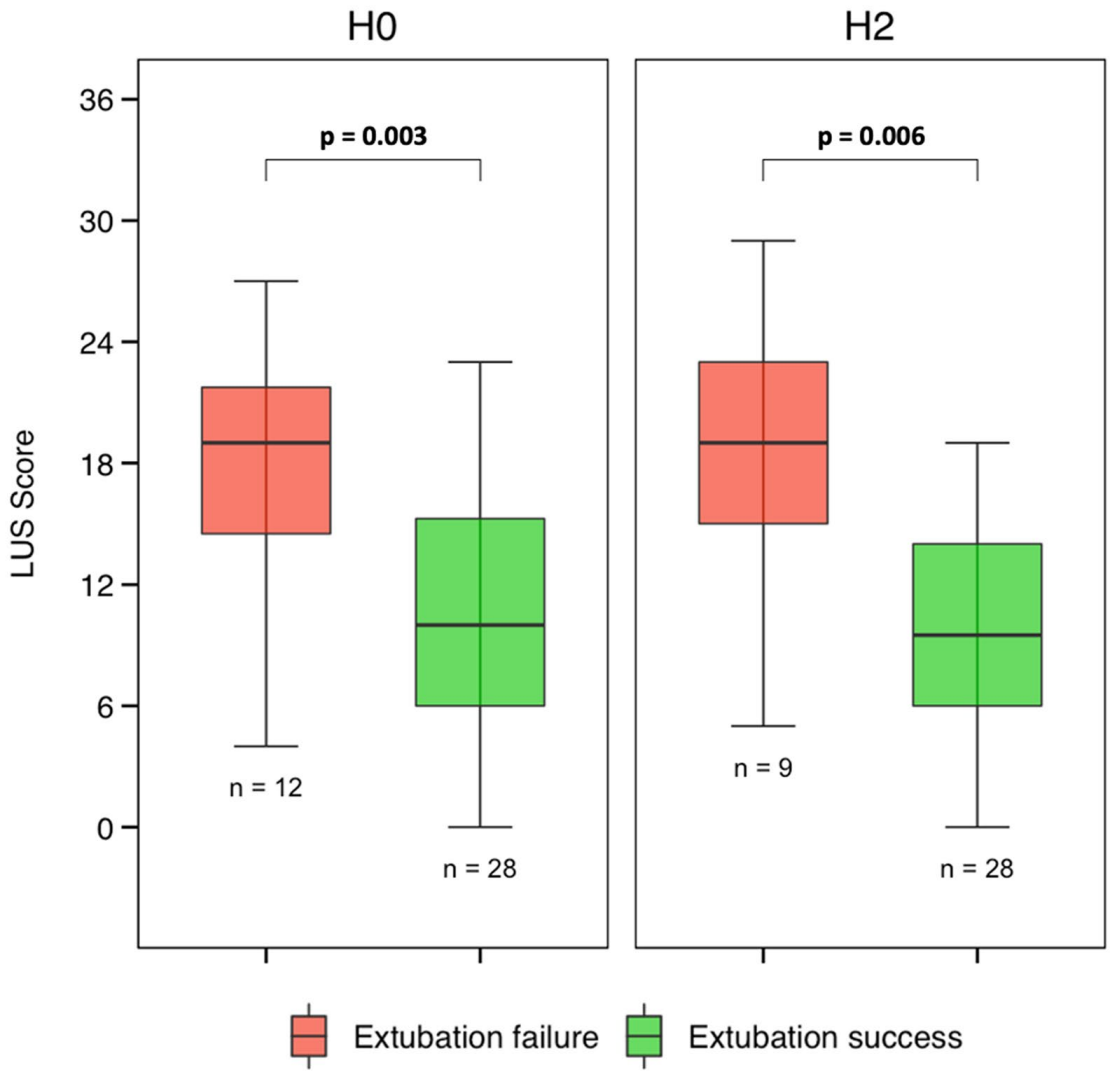
Changes of lung ultrasound score before and after extubation in patients with extubation failure and extubation success

Two hours after extubation, the LUS remains higher in the extubation failure group (19 vs 10, *p* = 0.006, Fig. 2) while echocardiography findings remained unchanged over time.

**Fig. 3:**
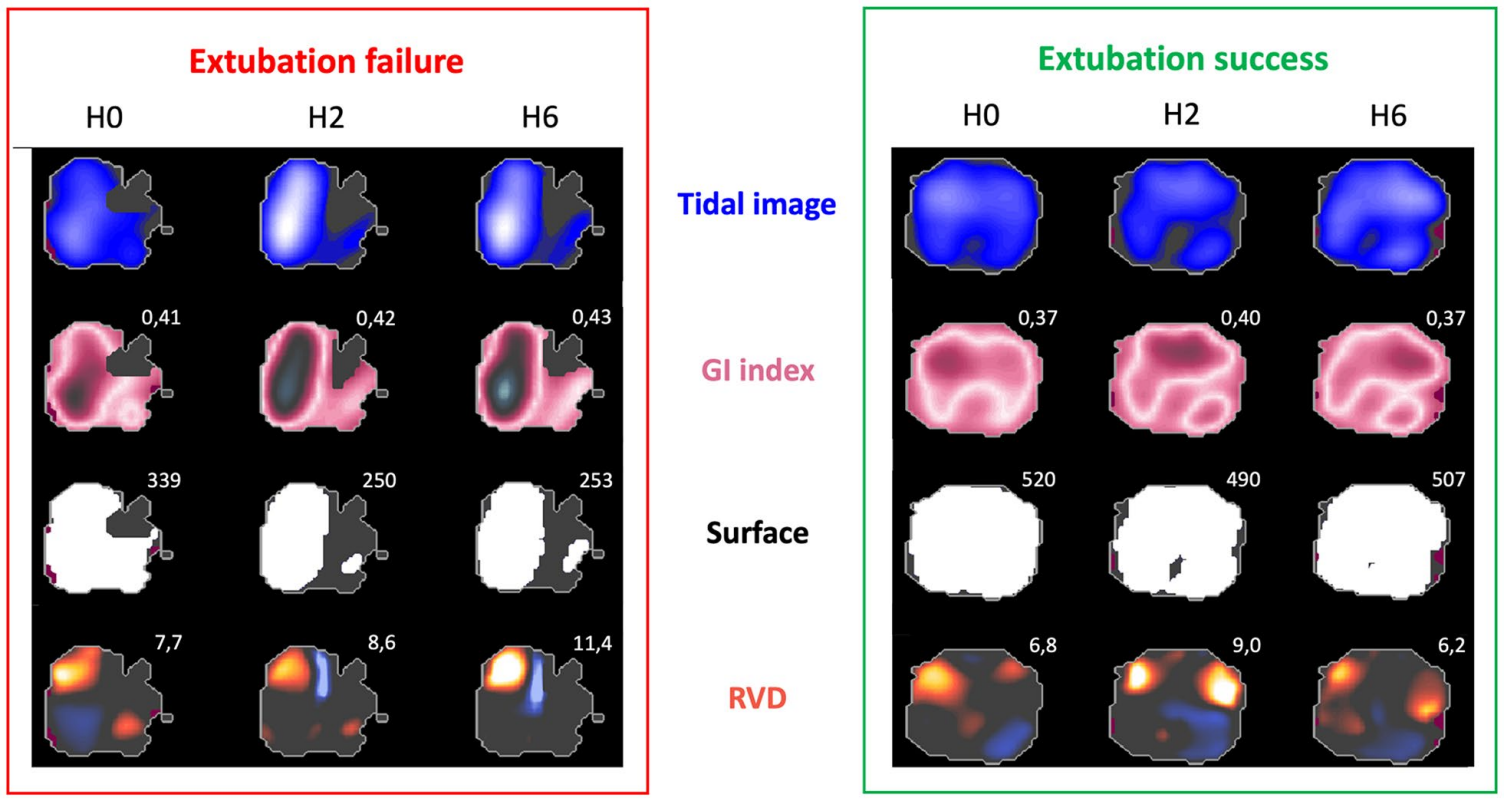
Example of EIT derived indices in a patient who experienced extubation failure and in a patient without extubation failure. The left panel corresponds to a patient who presented extubation failure 24 h after extubation due to a left atelectasis. The right panel corresponds to a patient without extubation failure. Values of EIT derived indices are given in white font for global inhomogeneity index (GI index), surface and regional ventilation delay (RVD)

A representative example of the time-course evolution of EIT derived indices is shown on Fig. 3.

**Fig. 4:**
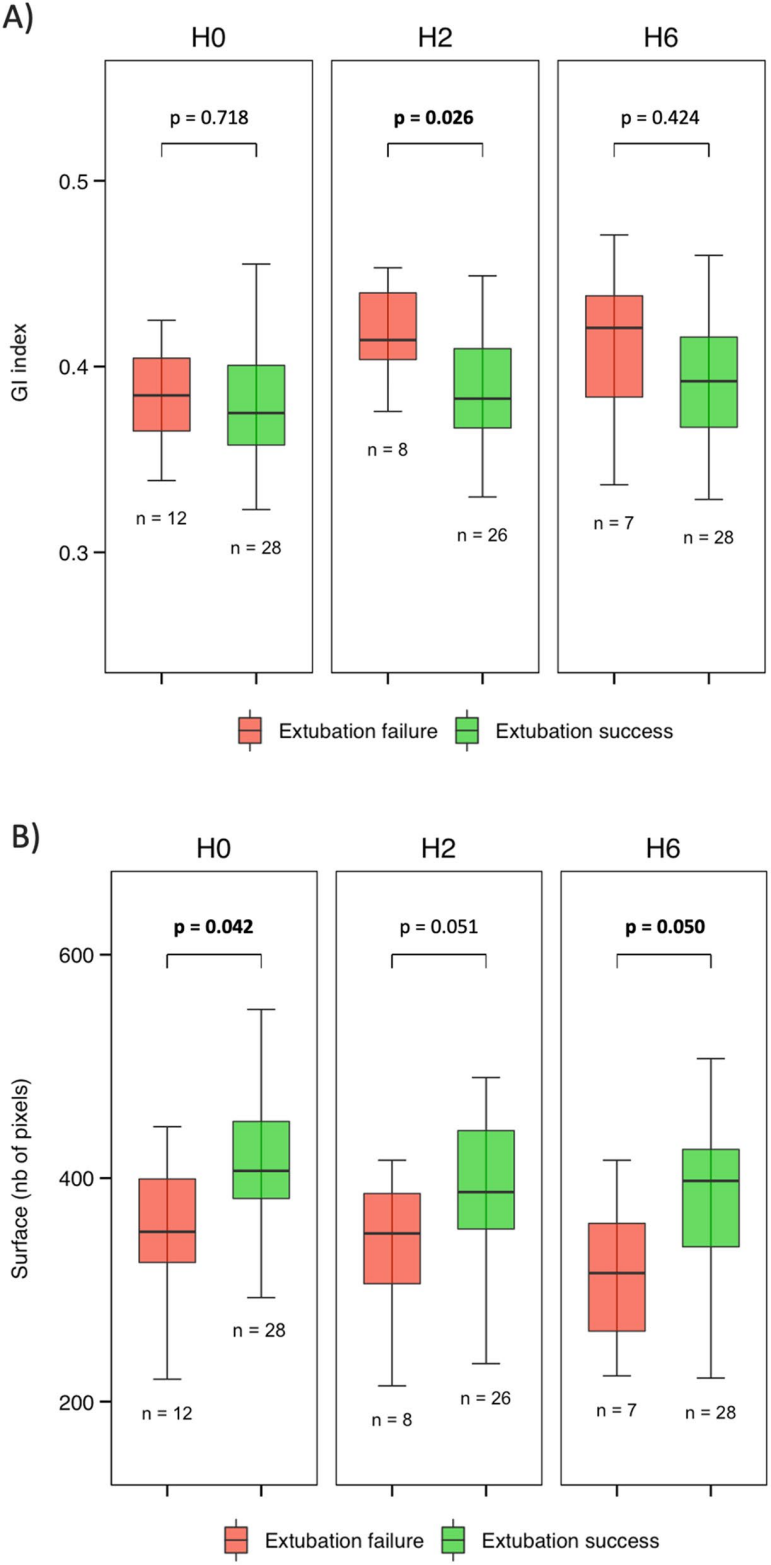
Time-course evolution of EIT derived indices before and after extubation, according to the extubation status. The top panel **A** corresponds to the global inhomogeneity index, the bottom panel **B** to the surface available for ventilation

After extubation and compared with patients who succeed extubation, patients who failed presented a higher GI index at H2 (0.41 vs 0.38, *p* = 0.026) and a smaller surface available for ventilation at H6 (315 vs 398 pixels, *p* = 0.050, Fig. 4). The center of ventilation and the RVD were not different between groups (see Additional file 2 and 3).

Analyses of the diagnostic performance of LUS, GI index and surface available for ventilation are described in Table 3.

**Table 3:** Threshold, area under the receiver operating characteristics curves (AUC-ROC), sensitivity, specificity, positive and negative predictive values of EIT derived indices and lung ultrasound score to predict extubation failure

	Threshold	AUC-ROC (95% CI)	Sensitivity (%) (95% CI)	Specificity (%) (95% CI)	Predictive values (%) (95% CI)	
					Positive	Negative
H0						
LUS	12	0.799 (0.642–0.957)	61 (43–70)	92 (75–100)	95 (83–100)	50 (39–60)
GI index	0.39	0.545 (0.346–0.744)	68 (50–82)	50 (16–75)	76 (64–88)	40 (20–60)
Surface	393	0.719 (0.543–0.894)	68 (50–85)	75 (50–100)	87 (75–100)	50 (36–69)
H2						
LUS	15	0.845 (0.657–1.000)	86 (71–96)	78 (44–100)	93 (83–100)	64 (44–89)
GI index	0.41	0.760 (0.572–0.947)	73 (54–88)	75 (41–100)	90 (79–100)	46 (29–70)
Surface	347	0.726 (0.524–0.928)	88 (73–100)	50 (13–88)	85 (77–96)	57 (25–100)
H6						
GI index	0.42	0.633 (0.353–0.913)	82 (68–96)	57 (14–86)	88 (79–96)	44 (18–75)
Surface	333	0.768 (0.571–0.965)	79 (64–93)	71 (29–100)	92 (83–100)	46 (25–71)

*LUS* lung ultrasound score, *GI* global inhomogeneity, *CI* Confidence interval

### Correlation between lung ultrasound and electrical impedance tomography

LUS was not correlated with EIT derived indices (see Additional file 4) but was correlated with baseline oxygenation (PaO_2_/FiO_2_ ratio, R^2^ = 0.230, *p* = 0.002).

## Discussion

This physiological study described lung aeration changes by regional lung ventilation with electrical impedance tomography and lung ultrasound in patients considered at high risk for extubation failure before and after extubation. The main results are the following: (1) before extubation, lung aeration (as estimated by the LUS and the surface available for ventilation) was lower in patients who subsequently developed extubation failure, as compared to patients who were successfully extubated; (2) after extubation, early changes in regional lung ventilation (surface available for ventilation and GI index) were observed in patients with extubation failure, whereas the LUS remains globally stable; (3) there was no correlation between LUS and EIT derived indices.

Respiratory causes represent at least 50% of all reasons of extubation failure [17] but clinical detection of respiratory worsening in early extubated patients is challenging since the monitoring of the respiratory function provided by the ventilator is no longer available. In a previous study, we reported that dyspnea assessment and respiratory muscles ultrasound within the two hours after extubation identify the patients who are likely to fail the extubation process [18]. It is noteworthy that patients who fail extubation for respiratory reasons are characterized by a loss in lung aeration [7, 19]. Therefore, assessment of lung aeration before and after extubation is potentially of clinical interest to identify high risk patients and implement timely and personalized preventive strategies such as noninvasive ventilation or high flow nasal oxygen. At the bedside, lung aeration can be assessed with lung ultrasound [20] and EIT [21]. While lung ultrasound provides a comprehensive lung aeration assessment at a given point [22], EIT is non operator dependent and provides a continuous assessment as do pulse oxygen saturation and cardio-monitoring.

Our study confirms previous data showing loss of lung aeration with lung ultrasound before extubation in patients failing extubation [7]. In addition, the surface available for ventilation was significantly lower in patients failing extubation. However, neither the center of ventilation, the GI index nor the regional ventilation delay were significantly different between extubation success and extubation failure. Mechanical ventilation with pressure support, before extubation, could tend towards a homogenization of the distribution of the ventilation. It is noteworthy that lung aeration as assessed by lung ultrasound was significantly different between extubation success and failure groups whereas except the surface available for ventilation, all EIT derived indices were similar before extubation. This may be explained by the fact that the GI index, the center of ventilation and the regional ventilation delay are qualitative descriptors of the heterogeneity in ventilation distribution, in contrast with the surface available for ventilation and the LUS that provide information on the quantity of ventilated area. In a previous study, Longhini et al. also found that the GI index was similar between extubation success and extubation failure patients before extubation (under pressure support ventilation) and that it increased significantly after extubation in the failure group [21]. In the former study, the GI index was monitored during the spontaneous breathing trial and in case of success, up to 30 min after the extubation. Another study using EIT during spontaneous breathing trial reported a decrease in regional tidal impedance variation likely related to lung derecruitment [21]. Our study is the first to explore the use of EIT after extubation. Our hypothesis was that EIT could detect changes in lung aeration shortly after extubation but that it could also be useful for a longer time. Indeed, we observed that after extubation, the GI index was significantly higher at H2, as well was the surface of available ventilation at H6 in patients who eventually failed the extubation as compared to their counterparts. The increase in GI and regional ventilation delay after extubation in the failure group may have several explanations in the context of extubation failure. The regional ventilation delay is a descriptor of regional recruitment inside the lung [23] and the GI quantifies the ventilation distribution, the higher the GI, the higher the inhomogeneity [13]. Therefore, our observations show that extubation failure is associated with inhomogeneous ventilation distribution. Bickenbach et al. suggested that the increase in GI and regional ventilation delay at the end of a spontaneous breathing trial may be related to a decrease in tidal volume or a decrease in lung compliance [24]. This particular pattern could also be explained by respiratory muscles weakness, in particular in case of diaphragm dysfunction at the time of weaning [19].

### Limitations

Our study has several limitations. First, it is an exploratory study with a limited sample size, our findings therefore warrant confirmation. Second, due to technical and logistical issues (signal artefacts, patients’ movement or cough, obesity, patients’ refusal), not all planned EIT recordings could be collected. Third, we did not analyze the tidal impedance variation that is an index of alveolar recruitment [25]. Reliable acquisition and analysis of this index requires to maintain the EIT belt attached to the patients during the period of the study, which was not accepted by our patients. Fourth, except for the evaluation of the cough strength, we did not undertake a comprehensive evaluation of the causes of extubation failure which would have required a more complex and less feasible protocol. Finally, only 58% of the patients received prophylactic noninvasive ventilation whereas they were all at high risk for extubation failure, mainly due to neurological disorders that limited NIV use.

## Conclusion

Chest electrical impedance tomography and lung ultrasound helped to identify patients at a very high risk of extubation failure, with preextubation loss of aeration and postextubation heterogeneity in air distribution.

### Supplementary Information

**Additional file 1.** Number of patients at each study’s visit.

**Additional file 2.** Time-course evolution of EIT derived indices (Regional ventilation delay and Center of Ventilation) before and after extubation, according to the extubation status. The top panel (A) corresponds to the Regional Ventilation Delay, the bottom panel (B) to the Center of Ventilation.

**Additional file 3.** Changes in lung aeration, regional lung ventilation and cardiac function before and after extubation, according to the extubation outcome.

**Additional file 4.** Correlations between LUS and EIT derived indices (all data from H0 and H2 pooled together).

## Data Availability

The datasets used and/or analyzed during the current study are available from the corresponding author on reasonable request.

## Abbreviations

ARDS: Acute respiratory distress syndrome
ARF: Acute respiratory failure
BMI: Body mass index
CoV: Center of ventilation
ECMO: Extracorporeal membrane oxygenation
EIT: Electrical impedance tomography
FiO_2_: Inspired fraction of oxygen
GI index: Global inhomogeneity index
HFNO: High-flow nasal oxygenation
IC-RDOS: Intensive care–respiratory distress observation scale
LUS: Lung ultrasound score
LVEF: Left ventricle ejection fraction
MV: Mechanical ventilation
NIV: Invasive ventilation
PaCO_2_: Arterial partial pressure of carbon dioxide
PaO_2_: Arterial partial pressure of oxygen
PEEP: Positive End Expiratory Pressure
RVD: Regional ventilation delay
SAPS II: Simplified acute physiology score 2
SBT: Spontaneous breathing trial
SOFA: Sequential Organ Failure Assessment
Vt: Tidal volume
VTI: Velocity time integral
