# Supplementary material for: Lung aeration estimated by chest electrical impedance tomography and lung ultrasound during extubation
Source: Ann Intensive Care. 2023 Sep 26;13:91. doi: 10.1186/s13613-023-01180-3 (PMC10522557; doi:10.1186/s13613-023-01180-3)
Supplement: Supplementary file 1 — Additional file 1. Number of patients at each study’s visit. [file 13613_2023_1180_MOESM1_ESM.pdf]

Additional file 1.

Number of patients at each study's visit.

	H0	H2	H6	H12	H24	H36	H48
Extubation success (n)	28	28	28	23	26	15	20
Extubation failure (n)	12	11	9	6	6	3	-
