# Supplementary material for: Lung aeration estimated by chest electrical impedance tomography and lung ultrasound during extubation
Source: Ann Intensive Care. 2023 Sep 26;13:91. doi: 10.1186/s13613-023-01180-3 (PMC10522557; doi:10.1186/s13613-023-01180-3)

Additional file 2.

Time-course evolution of EIT derived indices (Regional ventilation delay and Center of Ventilation) before (H0) and after extubation (H2, H6), according to the extubation status. The top panel (A) corresponds to the Regional Ventilation Delay, the bottom panel (B) to the Center of Ventilation.

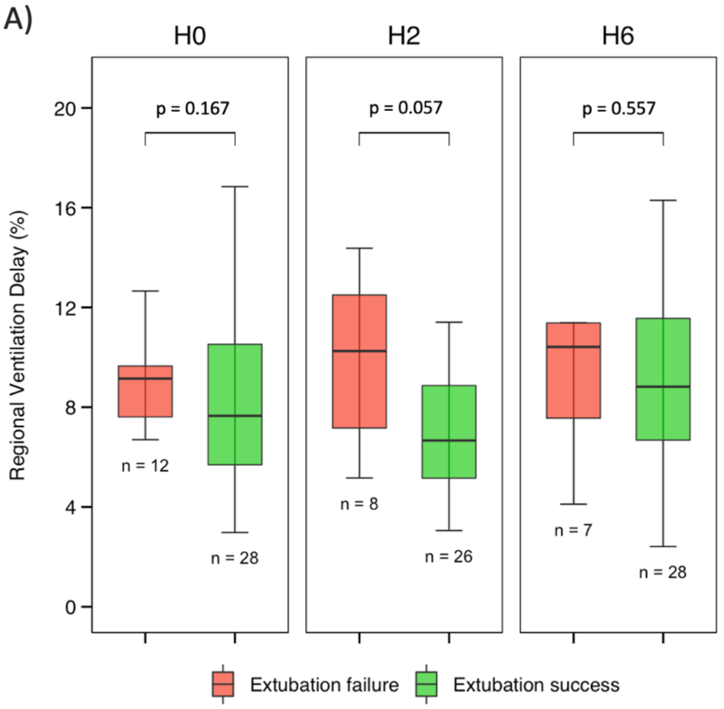

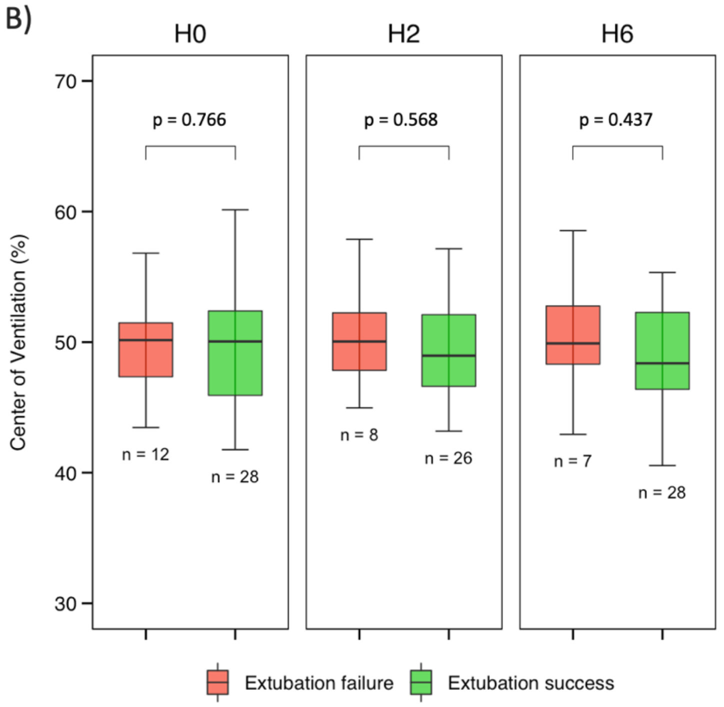

Supplement: Supplementary file 2 — Additional file 2. Time-course evolution of EIT derived indices (Regional ventilation delay and Center of Ventilation) before and after extubation, according to the extubation status. The top panel (A) corresponds to the Regional Ventilation Delay, the bottom panel (B) to the Center of Ventilation. [file 13613_2023_1180_MOESM2_ESM.pdf]
