# Supplementary material for: Lung aeration estimated by chest electrical impedance tomography and lung ultrasound during extubation
Source: Ann Intensive Care. 2023 Sep 26;13:91. doi: 10.1186/s13613-023-01180-3 (PMC10522557; doi:10.1186/s13613-023-01180-3)
Supplement: Supplementary file 3 — Additional file 3. Changes in lung aeration, regional lung ventilation and cardiac function before and after extubation, according to the extubation outcome. [file 13613_2023_1180_MOESM3_ESM.pdf]

Additional file 3.

Changes in lung aeration, regional lung ventilation and cardiac function before (H0) and after extubation (H2, H6), according to the extubation outcome

	**H0**	**H2**	**H6**
**Lung ultrasound**			
**Lung ultrasound score**			
Extubation success	10 (6 – 15)	10 (6 – 14)	-
Extubation failure	19 (14 – 22)	19 (15 – 23)	-
Success vs. failure	***p* = 0.003**	***p* = 0.006**	
**Posterior lung ultrasound score**			
Extubation success	5 (2 – 7)	5 (2 – 6)	-
Extubation failure	8 (7 – 10)	9 (6 – 11)	-
Success vs. failure	***p* < 0.001**	***p* = 0.007**	
**Electrical impedance tomography**			
**Global inhomogeneity index**			
Extubation success	0.37 (0.36 – 0.40)	0.38 (0.37 – 0.41)	0.39 (0.37 – 0.42)
Extubation failure	0.38 (0.37 – 0.40)	0.41 (0.40 – 0.44)	0.42 (0.38 – 0.44)
Success vs. failure	*p = 0.718*	***p = 0.026***	*p = 0.424*
**Center of ventilation (front-back), %**			
Extubation success	50 (46 – 52)	49 (47 – 52)	48 (46 – 52)
Extubation failure	50 (47 – 52)	50 (48 – 52)	50 (48 – 53)
Success vs. failure	*p = 0.766*	*p = 0.568*	*p = 0.437*

**Electrical impedance tomography**			
**Regional ventilation delay, %**			
Extubation success	7.7 (5.7 – 10.5)	6.7 (5.2 – 8.8)	8.8 (6.7 – 11.6)
Extubation failure	9.1 (7.6 – 9.7)	10.3 (7.2 – 12.5)	10.4 (7.6 – 11.4)
Success vs. failure	*p = 0.167*	*p = 0.057*	*p = 0.557*
**Surface, nb of pixels**			
Extubation success	406 (382 – 451)	388 (354 – 442)	398 (338 – 426)
Extubation failure	352 (324 – 399)	350 (306 – 386)	315 (263 – 360)
Success vs. failure	***p = 0.042***	*p = 0.051*	***p = 0.050***
**Cardiac function**			
**Left ventricle ejection fraction, %**			
Extubation success	45 (40 – 50)	50 (45 – 50)	-
Extubation failure	50 (48 – 58)	50 (45 – 52)	-
Success vs. failure	*p = 0.106*	*p = 0.702*	
**E/A**			
Extubation success	1.1 (0.9 – 1.2)	1.2 (0.9 – 1.4)	-
Extubation failure	1.2 (0.8 – 1.5)	0.8 (0.7 – 0.9)	-
Success vs. failure	*p = 0.792*	*p = 0.176*	
**E/E’**			
Extubation success	7.5 (6.6 – 9.9)	7.6 (6.9 – 8.7)	-
Extubation failure	8.6 (5.9 – 10.9)	7.9 (6.5 – 8.7)	-
Success vs. failure	*p = 0.973*	*p = 0.894*	

**Cardiac function**			
**Cardiac output, $L.min^{-1}$**			
Extubation success	7.1 (5.7 – 8.4)	7.1 (5.5 – 8.3)	-
Extubation failure	8.2 (7.0 – 8.9)	7.9 (7.0 – 9.4)	-
Success vs. failure	*p = 0.182*	*p = 0.327*	

E/A: early (E) over late (A) diastolic wave velocity ratio; E/e': E wave over tissue Doppler early (e') wave velocities at the lateral mitral valve annulus.
