# Supplementary material for: Lung aeration estimated by chest electrical impedance tomography and lung ultrasound during extubation
Source: Ann Intensive Care. 2023 Sep 26;13:91. doi: 10.1186/s13613-023-01180-3 (PMC10522557; doi:10.1186/s13613-023-01180-3)
Supplement: Supplementary file 4 — Additional file 4. Correlations between LUS and EIT derived indices (all data from H0 and H2 pooled together). [file 13613_2023_1180_MOESM4_ESM.pdf]

Additional file 4.

Correlations between LUS and EIT derived indices (all data from H0 and H2 pooled together)

	**Spearman coefficient**	**p-value**
Surface	$R^2 = 0.049$	$p = 0.059$
Global inhomogeneity index	$R^2 = 0.014$	$p = 0.326$
Regional ventilation delay	$R^2 = 0.006$	$p = 0.672$
Center of ventilation	$R^2 = 0.002$	$p = 0.518$
